# Role of Probiotics in *Mycoplasma pneumoniae* Pneumonia in Children: A Short-Term Pilot Project

**DOI:** 10.3389/fmicb.2018.03261

**Published:** 2019-01-09

**Authors:** Zongxin Ling, Xia Liu, Shu Guo, Yiwen Cheng, Li Shao, Dexiu Guan, Xiaoshuang Cui, Mingming Yang, Xiwei Xu

**Affiliations:** ^1^Collaborative Innovation Center for Diagnosis and Treatment of Infectious Diseases, State Key Laboratory for Diagnosis and Treatment of Infectious Diseases, The First Affiliated Hospital, School of Medicine, Zhejiang University, Hangzhou, China; ^2^Department of Gastroenterology, Affiliated Beijing Children’s Hospital, Capital Medical University, Beijing, China; ^3^School of Pharmacy, Shenyang Pharmaceutical University, Shenyang, China

**Keywords:** azithromycin, *Bifidobacterium*, *Clostridium*, *Mycoplasma pneumoniae* pneumonia, probiotics

## Abstract

*Mycoplasma pneumoniae* is one of the most common pathogens causing community-acquired pneumonia in children. *Mycoplasma pneumoniae* pneumonia (MPP) can be successfully treated with azithromycin; however, antibiotic-associated diarrhea (AAD) is a common adverse effect. Increasing evidence suggests that some probiotics may prevent the development of AAD. The present study determined the effects of probiotics (live *Clostridium butyricum* plus *Bifidobacterium infantis*) on the prevention and treatment of AAD in children with MPP when co-administered with intravenous azithromycin. Fifty-five children with MPP were enrolled and received azithromycin (10 mg/kg/day; once daily for 7 days) combined with probiotics (starting on the third day of azithromycin treatment; 1,500 mg three times daily); 50 healthy children served as controls. At the end of the trial, the incidence of AAD, fecal microbiota, intestinal mucosal barriers, and systemic inflammation were analyzed using recommended systems biology techniques. No cases of AAD or other adverse events occurred in children with MPP after co-administration of probiotics with azithromycin. A live *C. butyricum* plus *B. infantis* preparation partly reconstructed the gut microbiota, especially restoration of bacterial diversity. The indicators of intestinal mucosal barrier function, such as D-lactate, endotoxin, and diamine oxidase, were significantly improved and the systemic inflammation (interleukin 10) was attenuated after probiotic therapy. The present study indicated that co-administration of probiotics with azithromycin is a promising therapy for MPP treatment which could prevent and treat AAD effectively.

## Introduction

Community-acquired pneumonia (CAP) is the most common cause of death in children worldwide. Globally, CAP accounts for 15% of deaths in children <5 years of age and 922,000 deaths in children of all ages (Haq et al., 2017). Among all causative organisms, *Mycoplasma pneumoniae* (MP) is one of the most common pathogens causing CAP in children (Defilippi et al., 2008). *M. pneumoniae* pneumonia (MPP) not only causes pulmonary complications, such as bronchiolitis obliterans and necrotizing pneumonia, but also leads to a number of extra-pulmonary complications, such as encephalitis, arthritis, pericarditis, and hemolytic anemia, which can develop into severe life-threatening pneumonia. Due to the absence of a cell wall, MP is usually treated with antibiotics, such as quinolones, tetracyclines, and macrolides; however, only macrolides (erythromycin, azithromycin, clarithromycin, and roxithromycin) are used for children because of the potential side effects of alternative drugs, such as tetracyclines and fluoroquinolones (Youn and Lee, 2012). Azithromycin is generally the first-line treatment choice for MPP for children in our hospital because azithromycin is well-tolerated in the presence of a wide variety of concurrent illnesses and medications. It cannot be ignored, however, that the gastrointestinal adverse effects, such as diarrhea, are 72% higher with long-term azithromycin therapy (Florescu et al., 2009). Antibiotic-associated diarrhea (AAD) may be associated with dysbiosis of the gut microbiota that is disturbed by azithromycin (Xinli et al., 2013). Wei et al. (2018) reported that short-term azithromycin administration caused a 23% reduction in observed richness and 13% reduction in Shannon diversity. Recent studies have shown that the gut microbiota plays vital roles in numerous aspects of normal host physiology, from nutritional status to behavioral and stress responses (Subramanian et al., 2014; Jiang et al., 2015). A previous study showed that the decrease in short-chain fatty acid (SCFA)-producing bacteria cannot prevent the overgrowth of some potentially pathogenic intestinal microbes such as *Shigella* and *Escherichia*, which are associated with the development of diarrhea (Clausen et al., 1991; De Filippo et al., 2010). Johnston et al. (2016) demonstrated that co-administration of antibiotics with probiotics is associated with lower rates of AAD compared with controls, without an increase in clinically important adverse events. Our previous study showed that SCFA-producing probiotic strains, such as *Clostridium butyricum, Bifidobacterium infantis*, and mixtures of *C. butyricum* and *B. infantis* can restore gut microbiota and attenuate systemic inflammation in mice with AAD (Ling et al., 2015). The effects of probiotics on the prevention and treatment of AAD in children with MPP co-administered antibiotics, however, have not been thoroughly investigated. The purpose of the present study was to evaluate the effects of probiotics (live *C. butyricum* plus *B. infantis*) on children with MPP who are simultaneously treated with intravenous azithromycin, which will provide new adjuvant therapy for MPP in clinical practice.

## Materials and Methods

### Recruitment of Subjects

From January 2015 to June 2017, a total of 55 children with a final diagnosis of MPP were admitted to the Department of Gastroenterology at the Affiliated Beijing Children’s Hospital in China; 50 age- and gender-matched healthy children served as controls. All patients had symptoms and signs indicative of pneumonia at the time of admission, including a fever (>37.5°C), cough, abnormal breath sounds on auscultation, and an abnormal chest X-ray. MP infection was confirmed in nasopharyngeal secretions and serum samples using PCR and ELISA (Wang et al., 2014). The following exclusion criteria were established: age <1 month or >14 years; refractory MPP based on the presence of persistent fever and clinically, as well as radiologic deterioration after azithromycin treatment for ≥7 days; use of antibiotics, probiotics, prebiotics, or synbiotics in the previous month; and other respiratory tract infections, such as bacterial, fungal, chlamydial, or viral infections (such as respiratory syncytial virus, adenovirus, metapneumovirus, influenza virus A and B, and parainfluenza virus 1, 2, and 3); other diseases, such as asthma, chronic cardiac and pulmonary disease, rheumatic diseases, and immunodeficiency. The protocols for the present study were approved by the Ethics Committee of Affiliated Beijing Children’s Hospital at Capital Medical University (Beijing, China) and the methods were carried out in accordance with the approved guidelines (number: 2014-5). Written informed consent was obtained from the parents or guardians of all participants prior to enrollment.

### Treatment

All patients were treated with azithromycin intravenously at a dose of 10 mg/kg/day once daily for 7 days. On the third day of azithromycin treatment, the children were instructed to administer probiotics orally at least 2 h after antibiotic treatment until discharge from the hospital. A *C. butyricum* (CGMCC0313-1) combined with *B. infantis* (CGMCC0313-2) probiotic mixture (Changlekang^®^; Shandong Kexing Bioproducts Co., Ltd., Jinan, China) was used to treat the potential AAD. The freeze-dried probiotic mixture had >1.0 × 10^7^ CFU/g of viable *C. butyricum* and >1 × 10^6^ CFU/g of viable *B. infantis* per capsule. Children who were treated with probiotics received a dose of 1,500 mg three times daily.

### Sample Collection

On the third day of azithromycin treatment and the day of hospital discharge, fresh fecal samples (approximately 2 g) and blood samples were collected from each child for gut microbiota and intestinal mucosal barrier function analyses. These samples were transferred immediately to the laboratory and stored at -80°C after preparation within 15 min until use.

### Fecal Bacterial Genomic DNA Extraction

Bacterial genomic DNA was extracted using a QIAamp^®^ DNA Stool Mini Kit (Qiagen, Hilden, Germany) according to our previous study (Ling et al., 2015). The amount of bacterial genomic DNA was analyzed using a NanoDrop ND-1000 spectrophotometer (Thermo Scientific, Wilmington, DE, United States). The integrity and size of the bacterial genomic DNA were checked by electrophoresis. All bacterial genomic DNA were stored at -20°C for further use.

### Amplicon Library Construction and High-Throughput Sequencing

Amplicon libraries were constructed with Illumina sequencing-compatible and barcode-indexed bacterial PCR primers 319F/806R, which target the V3-V4 regions of the 16S rRNA gene (Fadrosh et al., 2014). All PCR reactions were performed with KAPA HiFi HotStart ReadyMix using the manufacturer’s protocol (Kapa Biosystems, Boston, MA, United States) and approximately 50 ng of extracted DNA per reaction. Thermocycling conditions were set at 95°C for 1 min, 55°C for 1 min, then 72°C for 1 min for 30 cycles, followed by a final extension at 72°C for 5 min. All PCR reactions were performed in 50 ml triplicates and combined after PCR. The amplicon library was prepared using a TruSeq^TM^ DNA sample preparation kit (Illumina, Inc., San Diego, CA, United States). Prior to sequencing, the DNA concentration of each PCR product was extracted with the MiniElute^®^ Gel Extraction Kit (Qiagen) and quantified on a NanoDrop ND-1000 spectrophotometer and Qubit 2.0 Fluorometer (Invitrogen, Carlsbad, CA, United States). The purified amplicons were then pooled in equimolar concentrations and the final concentration of the library was determined by Qubit. Negative DNA extraction controls (lysis buffer and kit reagents only) were amplified and sequenced as contamination controls. Sequencing was performed on a MiSeq instrument (Illumina, Inc.) using a 300 × 2 V3 kit together with PhiX Control V3 (Illumina, Inc.).

### Bioinformatic Analysis

The 16S rRNA gene sequence data sets generated from the MiSeq run were first merged and demultiplexed into per samples using QIIME (version 1.9.0) with default parameters (Caporaso et al., 2010). Chimera sequences were detected and removed using the USEARCH software based on the UCHIME algorithm (Edgar et al., 2011). An open-reference operational taxonomic unit (OTU) pick was then performed with USEARCH (version 7) referenced against the Greengenes database (version 13.8) at 97% sequence similarity (Edgar, 2010; Mcdonald et al., 2012), which was used for subsequent microbiota composition analysis. Alpha diversity was calculated using QIIME software with Python scripts based on the sequence similarity at the 97% level, including an index of observed species, abundance-based coverage estimator (ACE), Chao1 estimator, Shannon, Simpson, Evenness and PD whole tree. Sequence coverage was assessed in mothur by rarefaction curves and Good’s coverage (Good, 1953; Schloss et al., 2009). Beta diversity was measured by unweighted and weighted UniFrac distance calculated with 10 times of sub-sampling by QIIME. These distances were visualized by principal coordinate analysis (PCoA) with a different algorithm (Lozupone and Knight, 2005). Hierarchical clustering was performed and heatmap was generated using a Spearman’s rank correlation coefficient as a distance measure and a customized script developed in the R statistical package. Characterization of microorganismal features differentiating the gut microbiota was performed using the linear discriminant analysis (LDA) effect size (LEfSe) method^1^ for biomarker discovery, which emphasizes both statistical significance and biological relevance (Segata et al., 2011).

### Intestinal Mucosal Barrier Function Analysis

The parameters of intestinal mucosal barrier function, such as D-lactate, endotoxin (LPS), and diamine oxidase (DAO), were detected using the dry chemical method of the Intestinal Mucosal Barrier Biochemical Index Analysis System (JY-DLT; Beijing Zhongsheng Jinyu Diagnostic Technology Co., Ltd., Beijing, China). The anti-inflammatory cytokine, interleukin 10 (IL-10), in serum was detected using a human IL-10 immunoassay kit (eBioscience, San Diego, CA, United States). The data are presented as the mean ± standard deviation (SD) and the differences between the two groups were evaluated by Student’s *t*-test using SPSS (version 20.0; SPSS, Inc., Chicago, IL, United States).

### Statistical Analysis

Statistical analysis was performed using SPSS (version 20.0). GraphPad Prism (version 6.0; San Diego, CA, United States) was used for preparation of graphs. For bacterial diversity indices, bacterial composition at different taxonomic levels and parameters of intestinal mucosal barrier function, Student’s *t*-test and Mann–Whitney *U* test were applied. All tests of significance were two-sided, and a *p* < 0.05 was considered statistically significant for all analyses.

### Accession Number

The sequence data from this study are deposited in the GenBank Sequence Read Archive with the accession number SRP171107.

## Results

### General Information of Participants and Sequencing Data

A total of 55 children who were confirmed to have MPP were enrolled in the present study. Fifty age- and gender-matched healthy children were recruited as controls. The ages of both patients and healthy control subjects ranged from 3 to 6 years, with a female-to-male ratio of 0.58 in the MPP group (32/23) and 0.62 (31/19) in the control group. After successful treatment with azithromycin and probiotics, no AAD and other adverse events were observed in the MPP children. The average length of stay for these patients was 12.3 days (range, 11–14 days), while the average time for probiotic administration was 8.5 days (range, 8–11 days). On the third day of azithromycin treatment and the day of hospital discharge, feces and blood samples were collected from 55 patients; identical samples were also collected from the healthy children.

Based on the DNA amount and quality appropriate for 16S rRNA gene amplification and sequence analysis, 41 samples on the third day of azithromycin treatment (pre-treatment), 40 samples on the day of discharge (post-treatment) and 47 samples from healthy controls (control) were used for microbiota and intestinal mucosal barrier function analyses. Using the Illumina MiSeq sequencing platform, we generated 6,461,307 16S rRNA gene sequences from 128 samples, with an average sequence length of 438 nt, following paired-end merging and trimming. The average sequence depth per sample was 42,045 (minimum = 26,139; maximum = 62,103). All libraries had a Good’s coverage score ≥ 99.9% at the rarefaction point of 26,139 sequences, indicating that deep sequence coverage of the fecal microbiome was achieved for each sample. Thus, a total of 1,837,836 sequences were obtained from healthy controls for downstream analysis, while 1,819,648 sequences (pre-treatment) and 1,724,335 sequences (post-treatment) were obtained from children with MPP.

### Alterations in the Overall Structure of Fecal Microbiota After Probiotic Treatment

**FIGURE 1:**
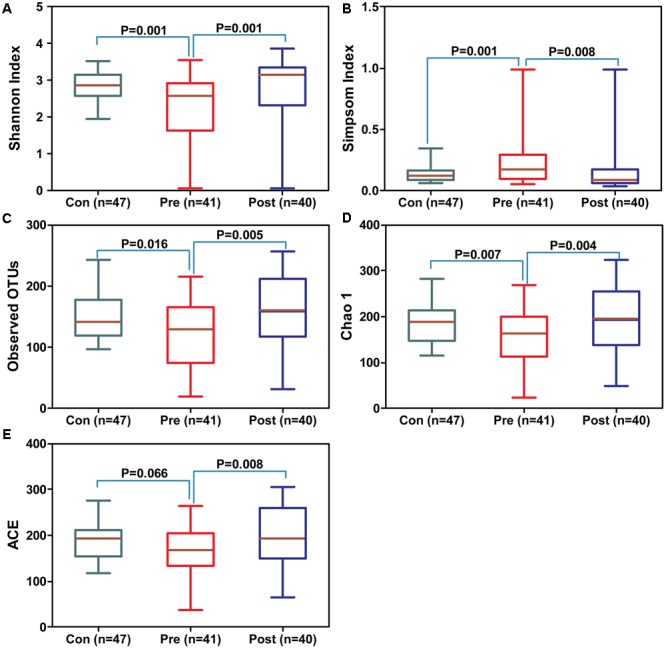
Structural alterations of the fecal microbiota after probiotic intervention for patients with *Mycoplasma pneumoniae* pneumonia (MPP). Diversity indices, such as Shannon **(A)** and Simpson **(B)**, and richness indices, such as observed species **(C)**, Chao 1 **(D)**, and ACE **(E)** were used to evaluate the overall structure of the fecal microbiota after probiotic therapy. The data are presented as the mean ± standard deviation. Unpaired *t*-tests (two-tailed) were used to analyze variation among the fecal microbiota.

**FIGURE 2:**
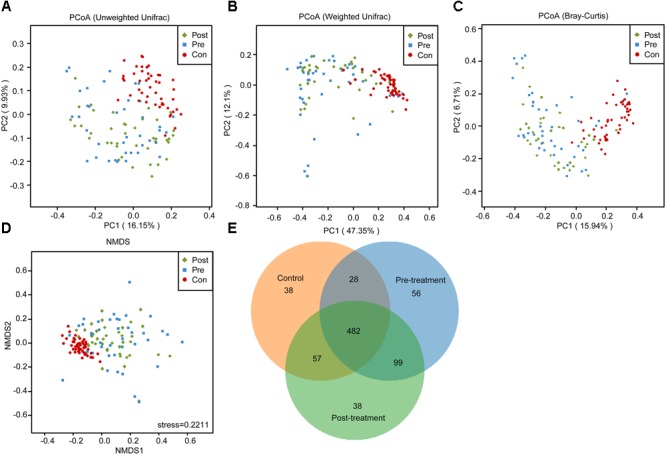
Beta-diversity analysis of the fecal microbiota in patients with *Mycoplasma pneumoniae* pneumonia after probiotic therapy. Principal coordinate analysis (PCoA) plots of individual fecal microbiota based on unweighted UniFrac distance **(A)**, weighted UniFrac distance **(B)**, Bray–Curtis distance **(C)** and non-metric multidimensional scaling (NMDS) analysis (**D**, stress = 0.2211). Each symbol represents a sample. The Venn diagram illustrates the overlap of OTUs in the fecal microbiota among control and pre- and post-treatment groups **(E)**.

In the present study, the total number of unique sequences from the three groups was 798, and represented all phylotypes. Figure 1 shows the alterations in the overall structure of fecal microbiota among control and pre- and post-treatment groups. The alpha diversity indices, such as Shannon and Simpson, showed that the fecal microbiota in the pre-treatment group were significantly lower than the control and post-treatment groups (Figures 1A,B; *p* < 0.05); however, there was no apparent difference between the control and post-treatment groups. The richness estimators, such as observed OTUs, Chao1, and ACE, showed similar changing patterns (Figures 1C–E). Our data indicated that the richness estimators were significantly decreased in the pre-treatment group when compared with control and post-treatment groups (*p* < 0.05). No significant differences between the control and post-treatment groups were observed for the diversity and richness indices (*p* > 0.05). The diversity indices and richness estimators of the fecal microbiota suggested a tendency toward microbiota restoration after probiotic treatment. Beta diversity analysis, such as PCoA based on the unweighted UniFrac, weighted UniFrac, and Bray–Curtis distances, indicated that there was a significant distinct clustering in the fecal microbiota between control and MPP patients, and a similar clustering between the pre-treatment and post-treatment groups (Figures 2A–C). Furthermore, another beta diversity analysis, the non-metric multi-dimensional scaling (NMDS) analysis, also revealed a similar clustering of the microbiota composition with the PCoA analysis (Figure 2D). A Venn diagram indicated that children shared a core set of bacteria in fecal microbiota regardless of the health status (Figure 2E). Despite significant interindividual variability, three clusters were found in fecal microbiota of children using unweighted UniFrac, which indicated that fecal microbiota was divided into cluster I in healthy controls, while fetal microbiota was divided into clusters II and III in pre- and post-treatment groups (Supplementary Figure S1).

**FIGURE 3:**
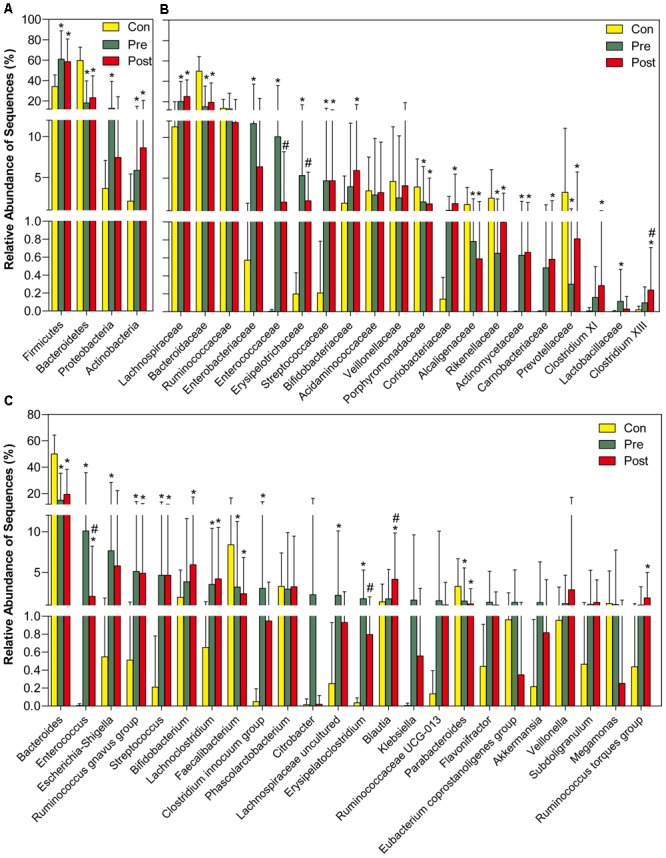
Different bacterial taxa among control and pre- and post-treatment groups. Comparisons of the relative abundance of abundant bacterial taxa at the level of bacterial phylum **(A)**, family **(B)**, and genus **(C)**. The data are presented as the mean ± standard deviation. Mann–Whitney *U*-tests were used to analyze variation among the three stomach microhabitats. ^∗^*p* < 0.05 compared with control group; ^#^*p* < 0.05 compared with pre-treatment group.

**FIGURE 4:**
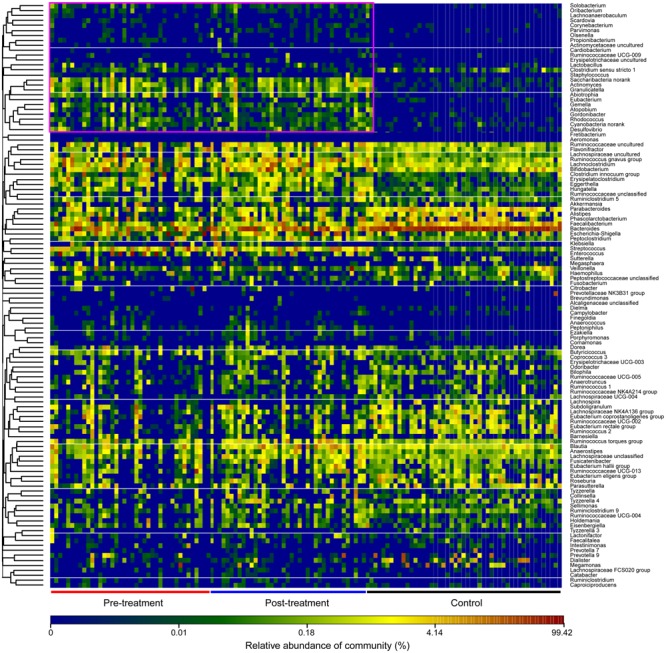
Heatmap of the key genera in the fecal microbiota among control and pre- and post-treatment groups. The color of the spots in the panel represents the relative abundance of the genus in each sample. The relative abundance of the bacteria in each genus is indicated by a gradient of color from green (low abundance) to red (high abundance). The genera were organized by Spearman’s correlation analysis based on relative abundances. The taxonomic classifications of the genus are shown on the right.

The sequences from the fecal microbiota could be classified as 17 phyla; however, the majority (≥95%) of sequences of the fecal microbiota were classified into four phyla, including Firmicutes, Bacteroidetes, Proteobacteria, and Actinobacteria (Figure 3A). Among the top four most abundant phyla in the fecal microbiota, Firmicutes and Actinobacteria were significantly increased and Bacteroidetes was decreased in the pre- and post-treatment groups when compared with the control group, while Proteobacteria was only increased in the pre-treatment group. Supplementary Figure S2 demonstrates the relative abundance of phyla in each sample among the three groups. At the family level, the relative abundance of Lachnospiraceae, Streptococcaceae, and Actinomycetaceae was significantly higher, while the relative abundance of Bacteroidaceae, Porphyromonadaceae, Alcaligenaceae, Rikenellaceae, and Prevotellaceae was clearly lower in both pre- and post-treatment groups when compared with the control group. A relatively higher abundance of Enterobacteriaceae, Enterococcaceae, Erysipelotrichaceae, and Lactobacillaceae was observed in the pre-treatment group, while a relatively higher abundance of Bifidobacteriaceae, Coriobacteriaceae, Carnobacteriaceae, and Clostridium XI existed in the post-treatment group (Figure 3B; *p* < 0.05). At the genus level, *Bacteroides, Faecalibacterium*, and *Parabacteroides* were significantly decreased, while *Enterococcus*, the *Ruminococcus gnavus* group, *Streptococcus*, and *Lachnoclostridium* were significantly increased in the pre- and post-treatment groups when compared with the control group. After probiotic treatment, we also showed that *Erysipelatoclostridium* was lower and *Blautia* was higher than in the pre-treatment group (Figure 3C; *p* < 0.05). Figure 4 shows a heatmap of bacterial genera in the pre- and post-treatment and control groups, which represented the relative percentages of the most abundant genera identified in each sample. Our data indicate that there were significant differences in the upper heatmap between the control and other two groups. Supplementary Figure S3 shows the relative abundance of genera in each sample among the three groups. LEfSe was used to compare the estimated phylotypes of the fecal microbiota among the three groups (Supplementary Figures S4–S7). In agreement with the previous analysis, opportunistic pathogens, such as *Enterococcus* and Enterobacteriaceae, were increased after antibiotic treatment, while butyrate-producing bacteria, such as *Clostridium* and *Ruminococcus*, were also increased after probiotic treatment. In summary, the diversity of the fecal microbiota in children with MPP showed a trend toward microbiota restoration after short-term probiotic treatment, yet there was no significant improvement in the composition of the fecal microbiota.

### Improvement in Intestinal Mucosal Barrier Function After Probiotic Treatment

**FIGURE 5:**
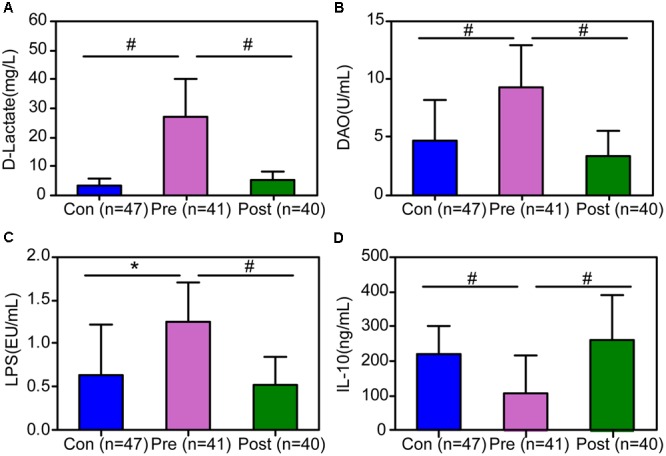
The improvement of the indicators of the intestinal mucosal barrier and anti-inflammatory interleukin 10 (IL-10) after probiotic treatment of children with MPP. The levels of D-lactate **(A)**, endotoxin (LPS) **(B)**, and diamine oxidase (DAO) **(C)** were decreased significantly after short-term probiotic treatment, **(D)** while the concentration of IL-10 was increased. The data are presented as the mean ± standard deviation (SD) and the differences between the two groups were evaluated by Student’s *t*-test. ^∗^*p* < 0.05; ^#^*p* < 0.01.

The intestinal mucosal barrier function was damaged in children with MPP after antibiotic treatment. The levels of D-lactate, DAO, and LPS were significantly increased, and the concentration of the anti-inflammatory cytokine, IL-10, was dramatically decreased (Figure 5). The increased parameters of intestinal mucosal barrier function, such as D-lactate, DAO, and LPS, represented the increased intestinal permeability, which has been reported to be related to bacterial translocation from the lumen to extra-intestinal sites. At the end of the trial, the parameters of intestinal mucosal barrier function and systemic inflammation were significantly improved after probiotic treatment. The present study indicated that a *C. butyricum* combined with *B. infantis* probiotic mixture repairs damaged intestinal mucosal permeability and attenuates systemic inflammation in children with MPP.

## Discussion

As a common pathogen of lower respiratory tract infections in children, *M*. *pneumoniae* accounts for up to one third of all cases of atypical CAP (Principi et al., 2001). With no cell wall, *M*. *pneumoniae* is resistant to beta-lactams and to all antimicrobials targeting the cell wall, but sensitive to macrolides and related antibiotics, including tetracyclines and fluoroquinolones. Due to the low toxicity, low MIC against the pathogen, and absence of a contraindication in young children, macrolides and related antibiotics, especially azithromycin, are usually the first-line treatment for MPP in children (Pereyre et al., 2016). The present study also showed that azithromycin successfully treated *M*. *pneumoniae* infection in children; however, the antimicrobial agents that we used against *M*. *pneumoniae* also disrupted co-evolved microbial communities that are integral to human health. Azithromycin led to microbiota dysbiosis and disturbed the colonization resistance of gastrointestinal microbiota, which induces clinical symptoms, most commonly diarrhea. A previous study has shown that the use of azithromycin is correlated with the increased incidence of AAD (Erdeve et al., 2004). Engelbrektson et al. (2009) demonstrated that a probiotic mixture consisting of *B. lactis* Bl-04, *B. lactis* Bi-07, *L. acidophilus* NCFM, *L. paracasei* Lpc-37, and *B. bifidum* Bb-02 minimizes the disruption of fecal microbiota in healthy subjects undergoing antibiotic therapy. Our previous data also indicated that a *C. butyricum* combined with *B. infantis* probiotic mixture restores fecal microbiota and attenuates systemic inflammation in mice with AAD (Ling et al., 2015). The present study determined the role of the abovementioned probiotic mixture in children with MPP treated with azithromycin.

In the present study, our data showed that there was no evident AAD for MPP children after azithromycin treatment with additional probiotic therapy. A previous study reported that azithromycin is most often associated with the development of AAD (Gorenek et al., 1999). Improvement of the therapeutic effects and reduction of side effects might be attributed to probiotic therapy, which was aimed at gut microbiota restoration. With high-throughput sequencing techniques, the diversity indices such as Shannon and Simpson indicated that the bacterial diversity of fecal microbiota showed clear trends in microbiota restoration, while the richness indices, such as observed OTUs and Chao1, demonstrated that the estimated phylotypes increased significantly after probiotic treatment. The overall structure of the fecal microbiota was reconstructed after azithromycin treatment when combined with probiotic therapy, which might be associated with the decreased incidence of AAD for children with MPP. Unfortunately, the composition of the fecal microbiota was not effectively restored with similar bacterial diversity after probiotic treatment. PCoA showed that the fecal microbiota could not be separated between antibiotic treatment and combined therapy, which might be related to short-term probiotic administration. Our previous study showed that the dysbiosis of the gut microbiota disturbed by antibiotics in mice did not recover quickly and naturally (Ling et al., 2015), although the native microbiota of the host displayed considerable resilience to the normal state after the antibiotic perturbation (Dethlefsen and Relman, 2011). Zaura et al. (2015) also reported that a single course of antibiotics is sufficient to disrupt the normal make-up of microorganisms in the gut for up to 1 year, potentially leading to antibiotic resistance (Jernberg et al., 2010). Based on our previous data, long-term probiotic therapy, but not short-term course, exerted beneficial effects on restoration of the gut microbiota (Ling et al., 2015). The present study was a short-term pilot clinical trial that evaluated the efficacy of probiotic therapy, which might affect the outcomes of microbiota restoration; however, short-term probiotic therapy was effective in improving clinical symptoms, ameliorating intestinal mucosal barrier function, and attenuating systemic inflammation. Despite successful clinical recovery, Song et al. (2013) also noted that the fecal microbiota of patients with recurrent *C. difficile* infection did not fully recover after fecal microbiota transplantation over 16 weeks. Rinne et al. (2006) also reported that probiotic intervention has short-term effects on gastrointestinal symptoms and long-term effects on gut microbiota. In agreement with the previous study, our data suggest that fecal microbiota restoration after probiotic intervention was slower than clinical recovery.

The probiotic mixture (live *C. butyricum* plus *B. infantis* preparation) has been used to modulate gut microbiota for children in China for many years, and can help achieve new eubiosis through supplementation of beneficial bacteria, stimulation of commensal bacteria growth, and consequent reduction of pathogenic species. Our previous studies have shown that the probiotic mixture, but not *C. butyricum* or *B. infantis* alone, exert beneficial effects on restoration of the gut microbiota, recovery of intestinal mucosal barrier function, and attenuation of systemic inflammation in animals and humans (Ling et al., 2015; Xia et al., 2018). Our animal study showed that the probiotic mixture helped restore the gut microbiota better than *C. butyricum* or *B. infantis* alone (Ling et al., 2015). *C. butyricum* (belonging to *Clostridium* cluster I) is a typical butyrate-producing, endospores-forming, Gram-positive obligate anaerobe that is isolated from soil and guts of healthy animals and humans. The longevity of endospores and the resistance to both chemical and physical stresses have determined the survival of *C. butyricum* at lower pH and relatively higher bile concentrations (Kong et al., 2011). These properties are a benefit of the use of *C. butyricum* as a probiotic in clinical practice. A previous study has shown that *C. butyricum* MIYAIRI 588 is effective for the treatment and the prophylaxis of AAD in children in Japan, as *C. butyricum* MIYAIRI 588 normalizes the gut microbiota disturbed by antibiotics (Seki et al., 2003). Previous studies have shown that *C. butyricum* promotes the growth of beneficial strains of *Lactobacillus* and *Bifidobacterium* and inhibits the harmful strain of *C. perfringens* (Seki et al., 2003; Kong et al., 2011; Zhang et al., 2011), which is consistent with our previous study on mice. Our previous study also showed that *C. butyricum* attenuates cerebral ischemia/reperfusion injuries in diabetic mice via modulation of gut microbiota, which regulates the bidirectional communication of the gut-brain axis (Sun et al., 2016). The potential underlying mechanism might be associated with elevated levels of SCFAs, especially butyrate after the administration of *C. butyricum*, which plays an important role in recovering intestinal tight junctions and maintaining gut integrity. *Bifidobacterium*, the dominant commensal bacteria of colonic microbiota, accounts for up to 25% of the cultivable fecal bacteria in adults and 80% in infants. Owing to the rare association with infection, *Bifidobacteria* have been studied for efficacy in the prevention and treatment of a broad spectrum of animal and/or human gastrointestinal disorders as probiotic strains. A previous study has shown that *B. infantis*, alone or in combination with other bacteria, can specifically relieve various symptoms of irritable bowel syndrome (Whorwell et al., 2006), and reduce the incidence and severity of necrotizing enterocolitis in very low birth weight infants (Lin et al., 2005). Charbonneau et al. (2013) demonstrated that supplementation with *B. infantis* potentially altered the composition of the fecal microbiota in patients with irritable bowel syndrome. The present study also showed that *Bifidobacterium* and *Clostridium* increased significantly after probiotic treatment. A previous study has found that secreted bioactive factors from *B. infantis*, such as SCFAs and peptides, retain their biological activity *in vivo*, and are effective in normalizing gut permeability and improving disease in an animal model of colitis (Ewaschuk et al., 2008). In agreement with our previous study on mice, our data indicated that live *C. butyricum* plus *B. infantis* preparation repaired the intestinal mucosal barrier function and significantly alleviated the host inflammatory response, even though probiotic therapy was administered relatively short-term. Chen et al. (2010) also reported that the short-term use of probiotics preparation, including live *C. butyricum*, not only improved the composition of the gut microbiota, but also attenuated the severity of acute diarrhea in hospitalized children and was associated with a reduced length of hospital stay; however, the efficacy of probiotics in treating infections and AAD has been recently questioned (Suez et al., 2018; Zmora et al., 2018) and some clinical studies have even reported probiotic-associated morbidity and mortality (Besselink et al., 2008; Vogel, 2008). Probiotics have been proposed to constitute an effective preventive treatment for antibiotic-induced dysbiosis; however, the adverse effects associated with probiotic consumption may be under-reported in clinical trials (Bafeta et al., 2018). Recent studies conducted by one group from Israel have demonstrated that post-antibiotic probiotic benefits may be offset by compromised gut mucosal recovery, and empiric probiotics supplementation may delay gut microbiome and transcriptome reconstitution post-antibiotic treatment (Suez et al., 2018; Zmora et al., 2018). They consider that probiotics treatment should be person-, strain-, and disease-specific, but not an empiric “one size fits all” probiotic regimen design. Even so, the available evidence does not completely refute the positive roles of probiotics. The health benefits conferred by probiotic bacteria are strain-specific, depending on the strain and disease tested (Whelan and Quigley, 2013). The present study has demonstrated that the two bacterial strains have shown very promising results, preventing the occurrence of AAD in children.

There were several limitations to the present study. First, the present study only observed the therapeutic effects of short-term probiotic intervention on children with MPP, while additional doses of the probiotics administered for longer durations are needed to further explore the role in gut microbiota restoration and relief of clinical symptoms for children discharged from the hospital with MPP. Second, the number of patients in the present study was small and the follow-up time was relatively short; children with MPP who are treated with probiotics with a longer duration of follow-up would strengthen our findings and conclusions. Third, the potential protective effects of a live *C. butyricum* plus *B. infantis* preparation to prevent AAD in children did not withstand intention-to-treat analysis. Future randomized controlled trials will be designed with probiotics and other types of gastrointestinal protective agents which can help determine the actual role and mechanism underlying these beneficial bacteria. Fourth, the possibility of natural recovery of gut microbiota in these children with MPP was not considered in the present clinical experimental design, although the placebo control helped to determine the role of the probiotics more accurately. Thus, the present report can be regarded as a pilot study that should be verified in further clinical trials.

## Conclusion

In conclusion, the present study has shown that short-term use of a live *C. butyricum* plus *B. infantis* preparation was effective in preventing the development of AAD in children hospitalized with MPP who were treated with azithromycin. The probiotic mixture partly reconstructed the gut microbiota, especially restoration of bacterial diversity, which might be due to short-term probiotic intervention. Interestingly, the intestinal mucosal barrier function was significantly improved and systemic inflammation was attenuated after probiotic therapy. Therefore, the administration of a live *C. butyricum* plus *B. infantis* preparation may become a promising therapy for prevention and treatment of AAD.

## Author Contributions

ZL and XX conceived and designed the study. ZL, XL, YC, and LS generated the sequencing data. XL, DG, SG, XC, MY, and XX collected the samples. ZL, XL, LS, and XX analyzed the data, carried out the computational analysis, interpreted the data, and drafted the manuscript.

## Conflict of Interest Statement

The authors declare that the research was conducted in the absence of any commercial or financial relationships that could be construed as a potential conflict of interest.

## Footnotes

1 http://huttenhower.sph.harvard.edu/lefse/

## Supplementary Material

FIGURE S1 Differentiation in the fecal microbiota from each sample of control and pre- and post-treatment groups (interpersonal variations). Community differentiation was measured using the unweighted UniFrac algorithm; the scale bar indicates the distance between clusters in UniFrac units. All of the branch nodes shown were significant (*p* < 0.001).

FIGURE S2 Comparison of the relative abundances of bacterial phyla of each sample among control and pre- and post-treatment groups.

FIGURE S3 Comparison of the relative abundances of bacterial genera of each sample among control and pre- and post-treatment groups.

FIGURE S4 Taxonomic differences of the fecal microbiota among control and pre- and post-treatment groups using LEfSe.

FIGURE S5 Taxonomic differences of the fecal microbiota between control and pre-treatment groups using LEfSe.

FIGURE S6 Taxonomic differences of the fecal microbiota between control and post-treatment groups using LEfSe.

FIGURE S7 Taxonomic differences of the fecal microbiota between pre- and post-treatment groups using LEfSe.
