# Supplementary figures and images for: Role of Probiotics in Mycoplasma pneumoniae Pneumonia in Children: A Short-Term Pilot Project
Source: Front Microbiol. 2019 Jan 9;9:3261. doi: 10.3389/fmicb.2018.03261 (PMC6334620; doi:10.3389/fmicb.2018.03261)

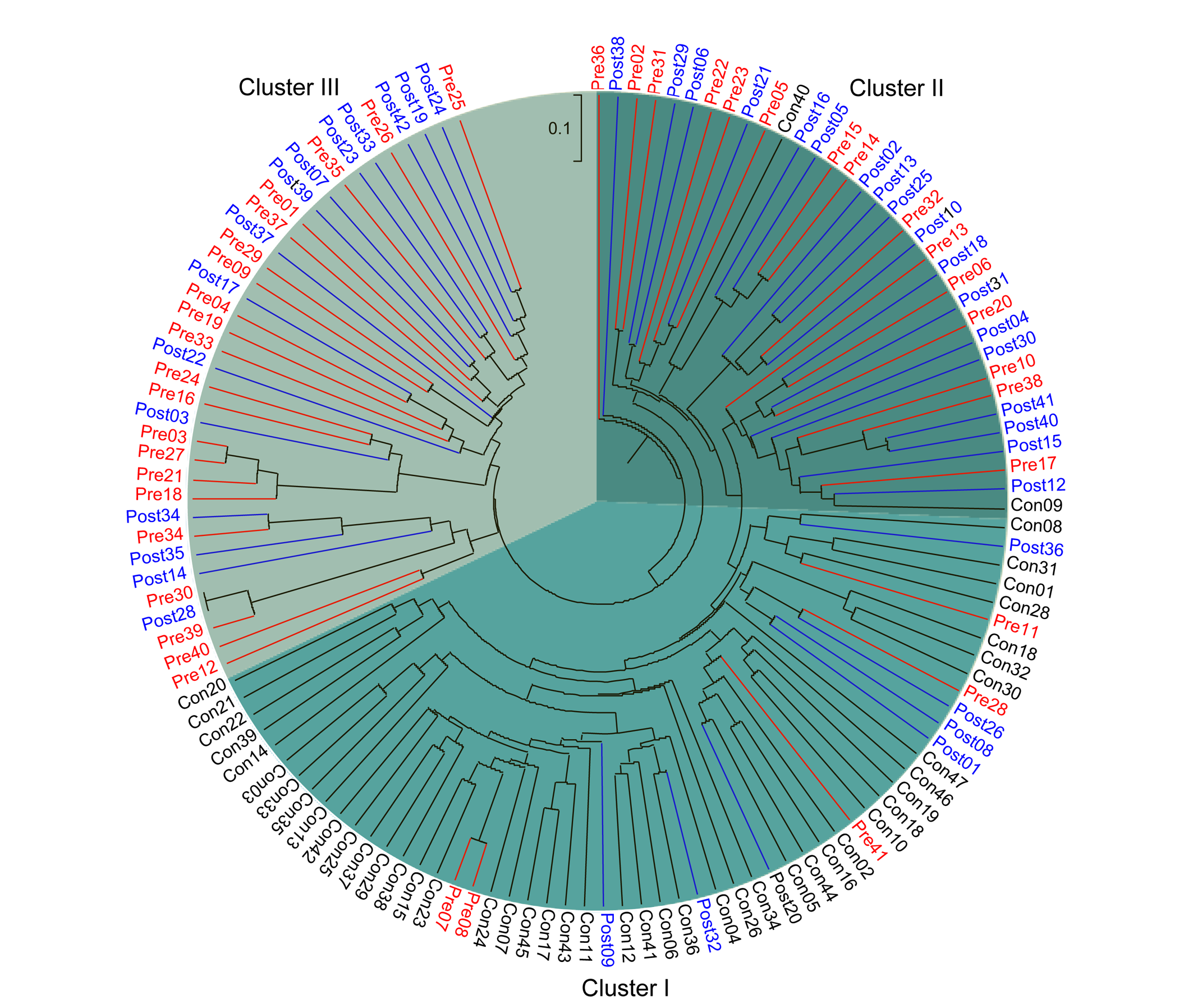

Supplement: FIGURE S1 — Differentiation in the fecal microbiota from each sample of control and pre- and post-treatment groups (interpersonal variations). Community differentiation was measured using the unweighted UniFrac algorithm; the scale bar indicates the distance between clusters in UniFrac units. All of the branch nodes shown were significant (p < 0.001). [file Image_1.TIF]

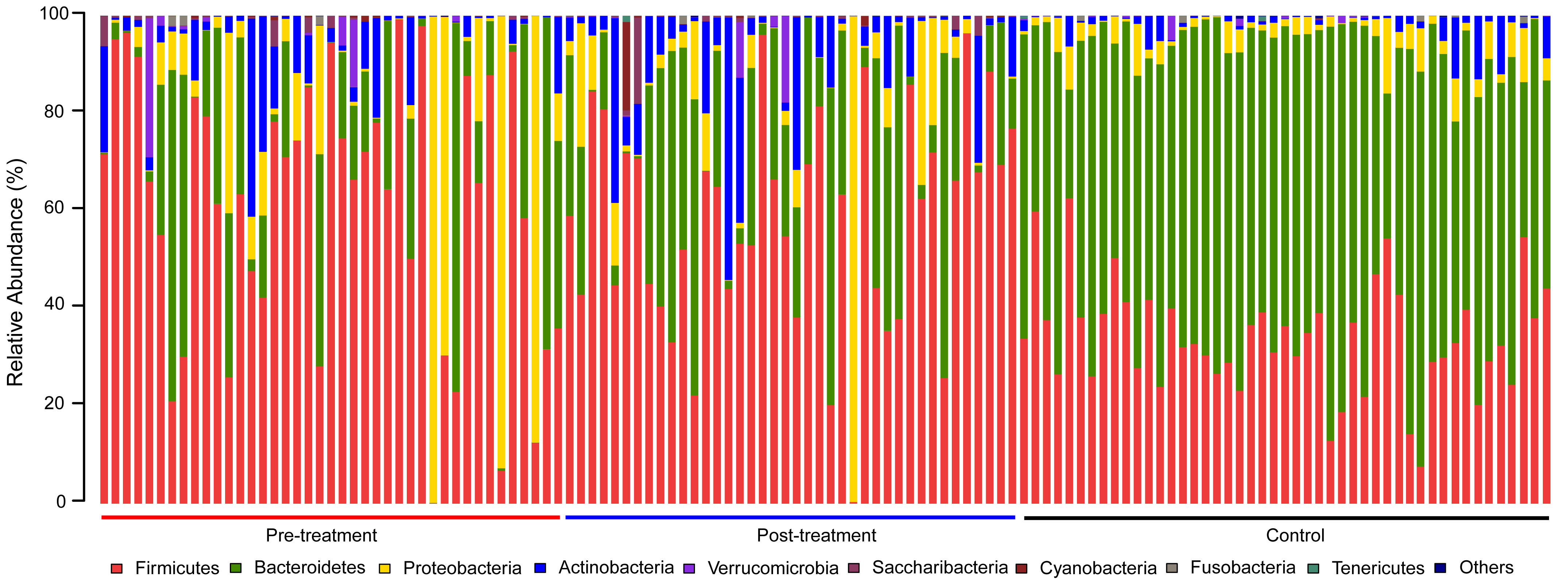

Supplement: FIGURE S2 — Comparison of the relative abundances of bacterial phyla of each sample among control and pre- and post-treatment groups. [file Image_2.TIF]

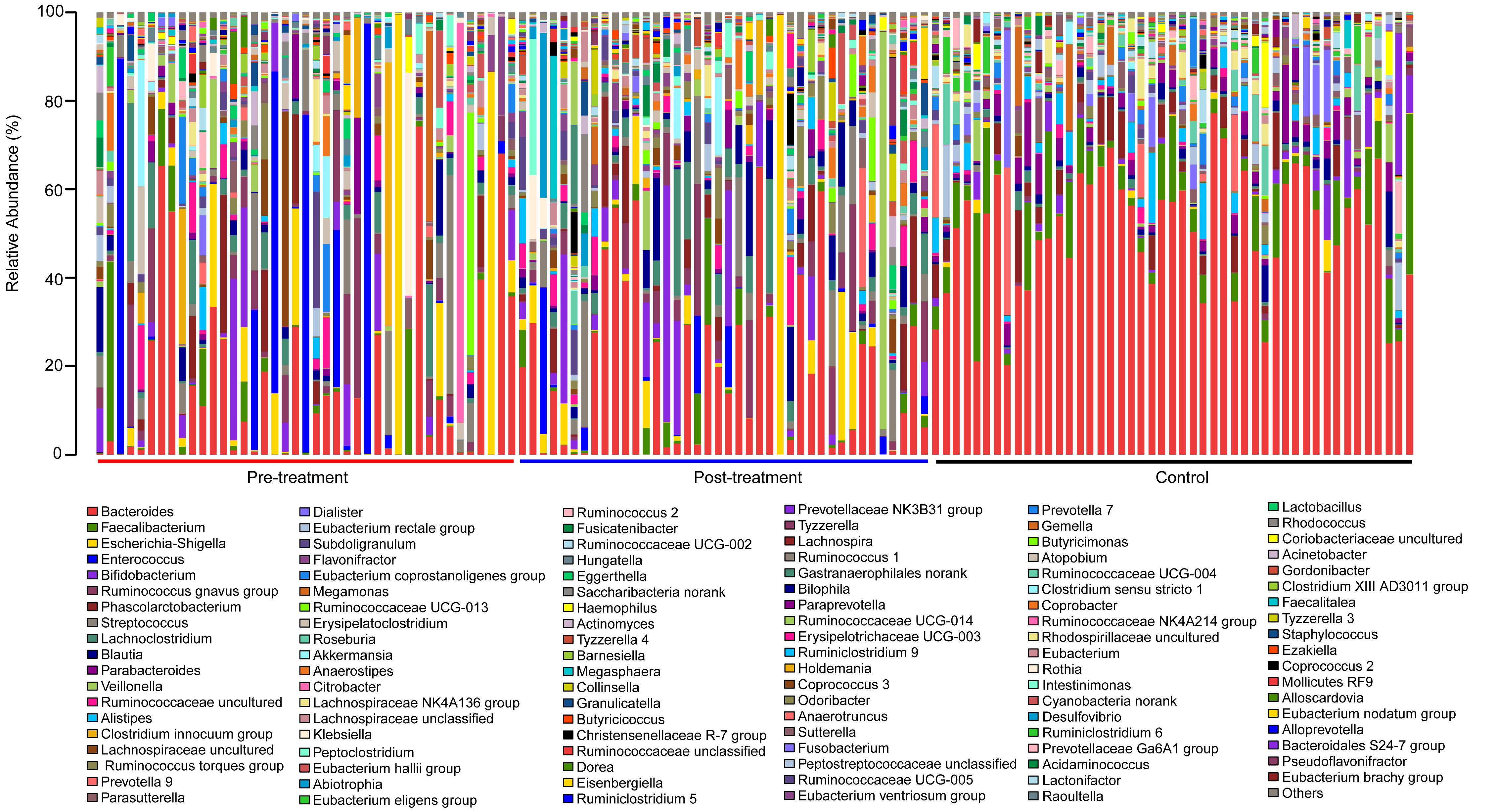

Supplement: FIGURE S3 — Comparison of the relative abundances of bacterial genera of each sample among control and pre- and post-treatment groups. [file Image_3.TIF]

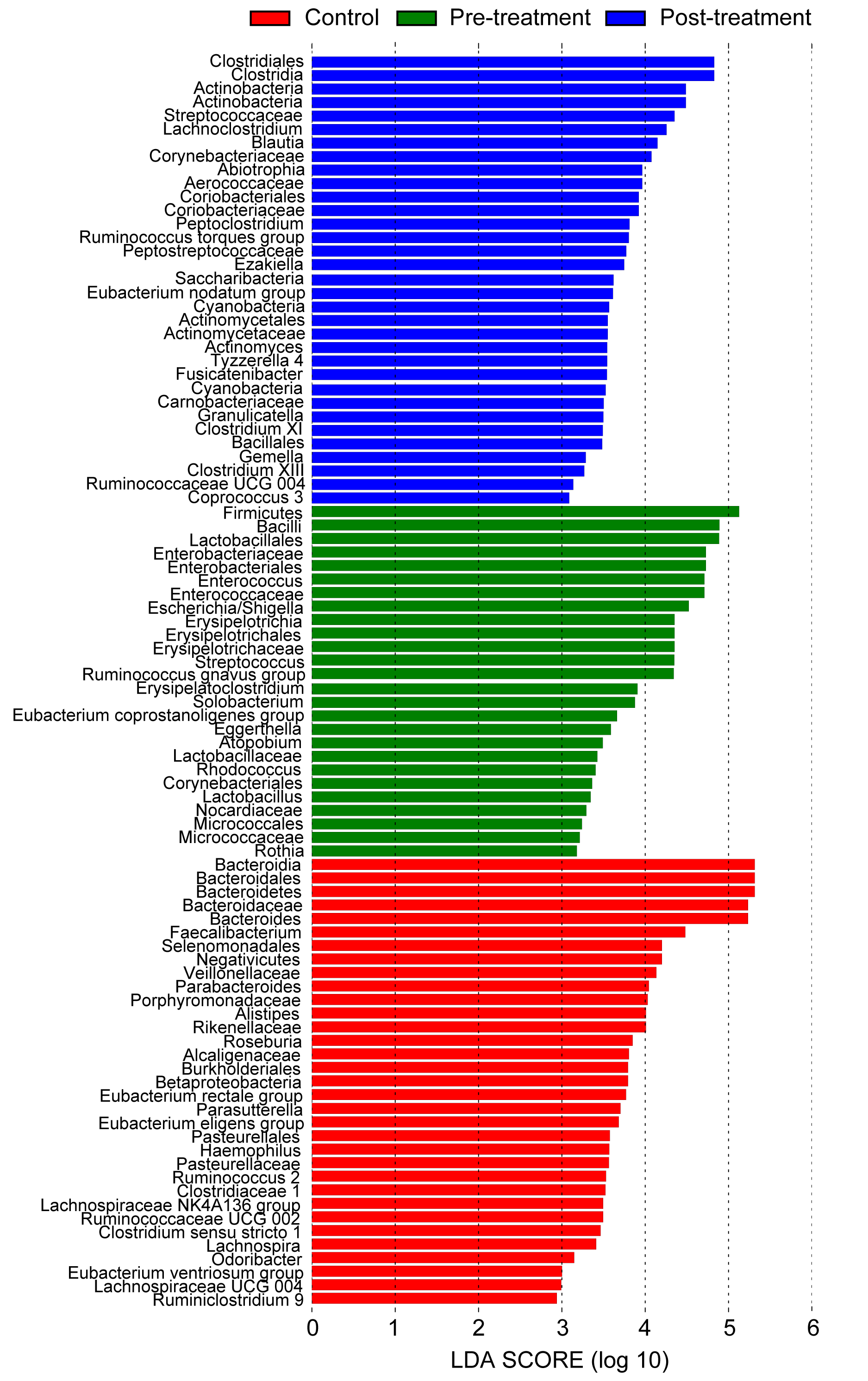

Supplement: FIGURE S4 — Taxonomic differences of the fecal microbiota among control and pre- and post-treatment groups using LEfSe. [file Image_4.TIF]

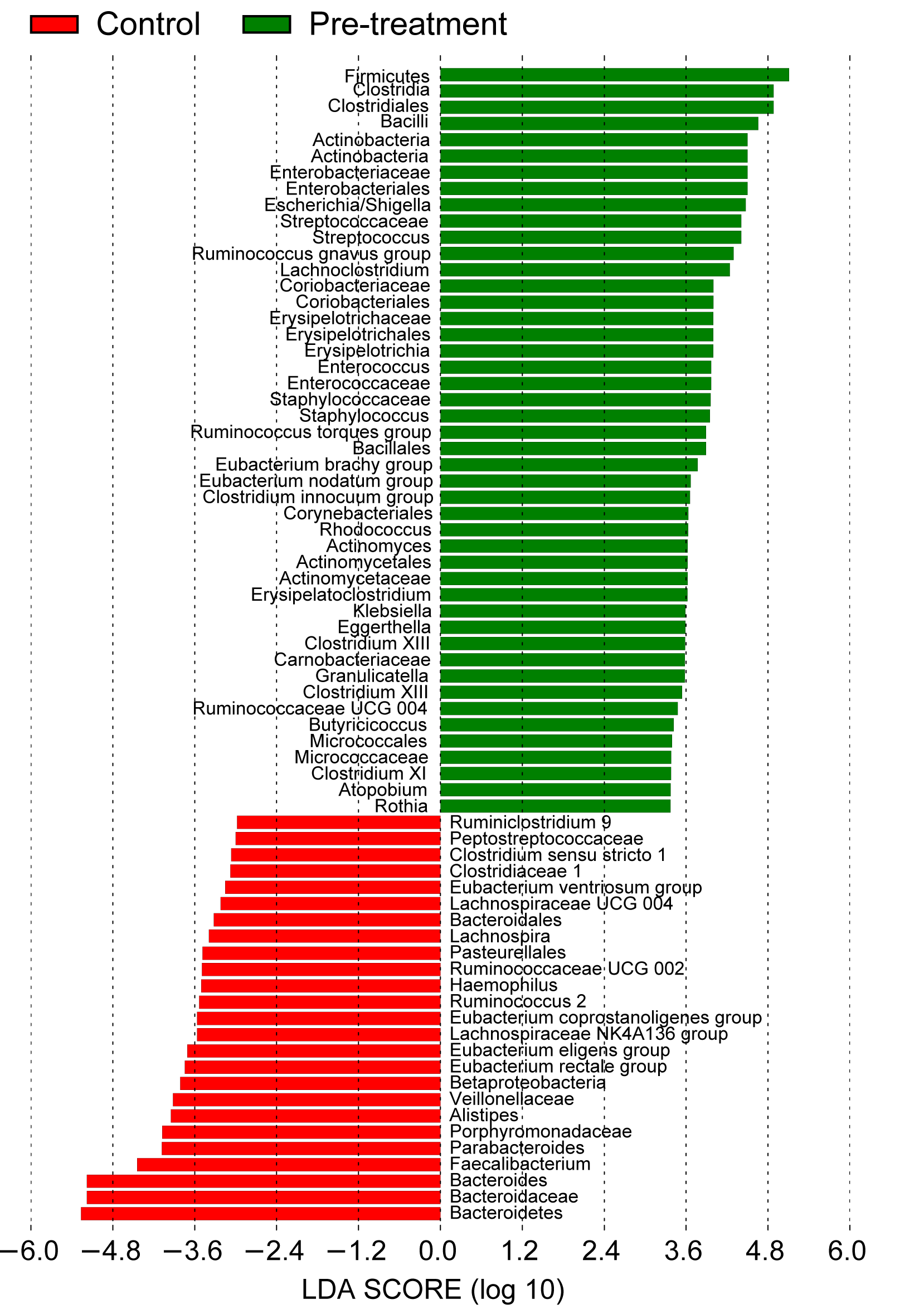

Supplement: FIGURE S5 — Taxonomic differences of the fecal microbiota between control and pre-treatment groups using LEfSe. [file Image_5.TIF]

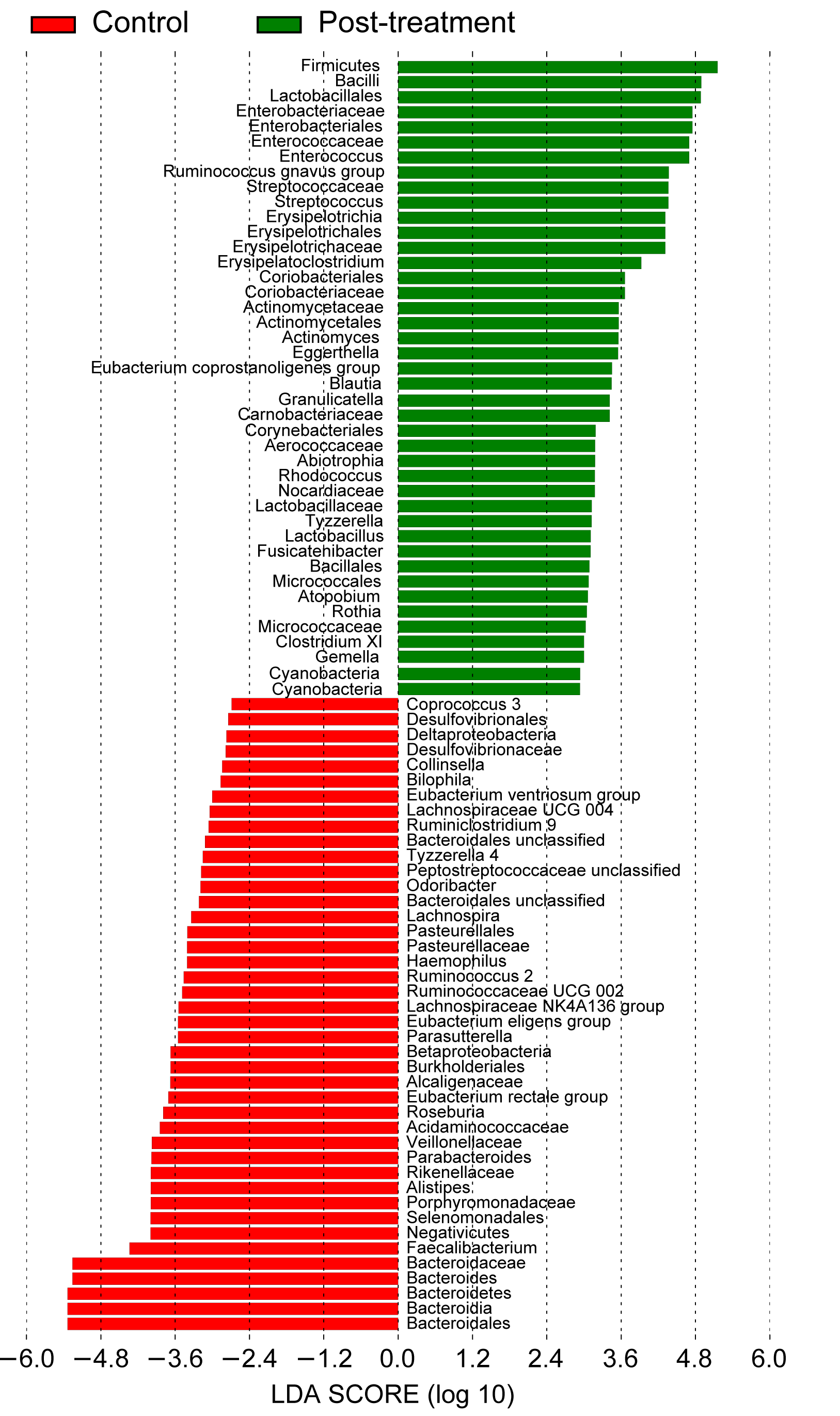

Supplement: FIGURE S6 — Taxonomic differences of the fecal microbiota between control and post-treatment groups using LEfSe. [file Image_6.TIF]

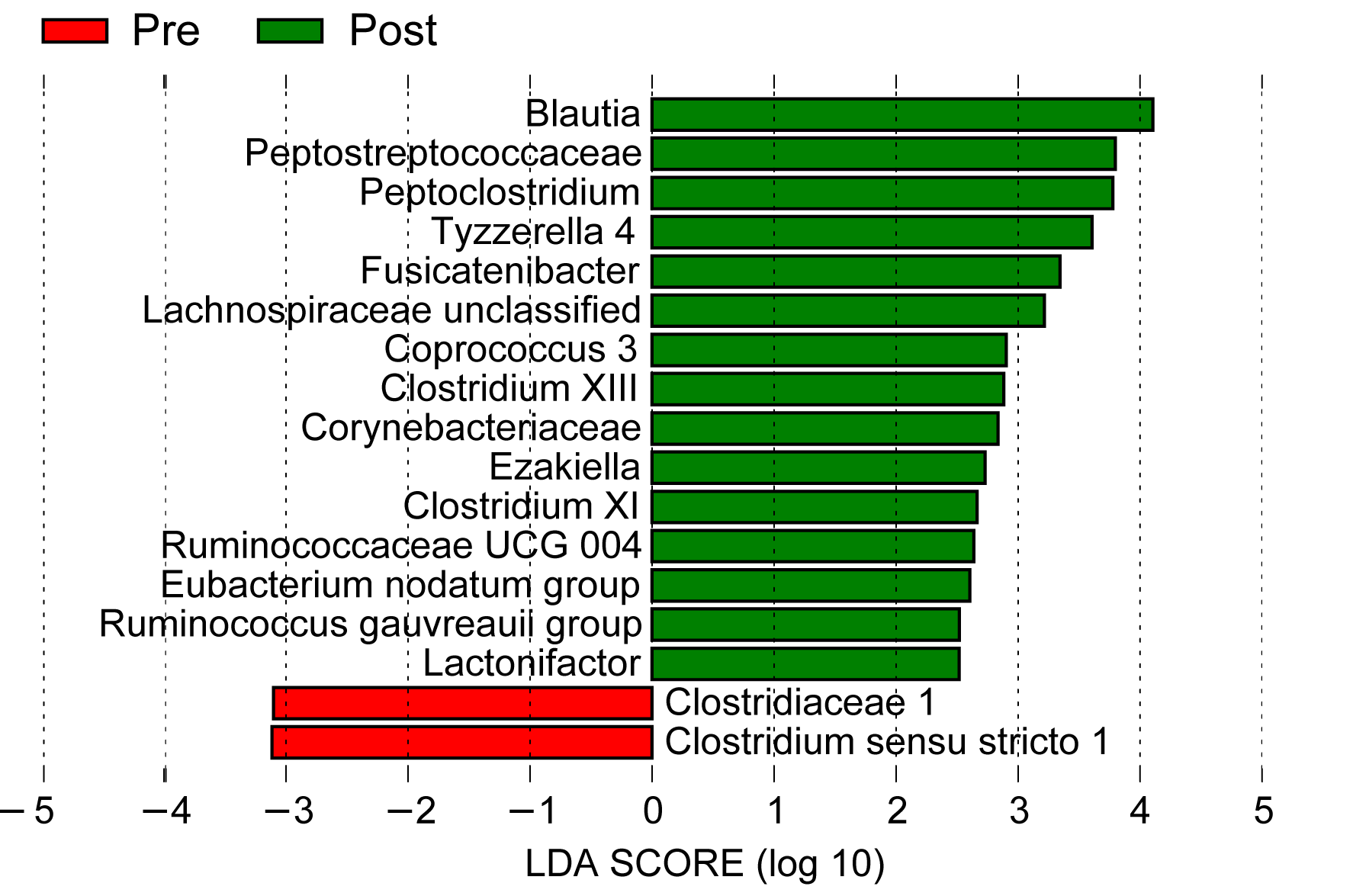

Supplement: FIGURE S7 — Taxonomic differences of the fecal microbiota between pre- and post-treatment groups using LEfSe. [file Image_7.TIF]
